# Antifeedant Activity of *Ginkgo biloba* Secondary Metabolites against *Hyphantria cunea* Larvae: Mechanisms and Applications

**DOI:** 10.1371/journal.pone.0155682

**Published:** 2016-05-23

**Authors:** Long Pan, Lili Ren, Fang Chen, Yuqian Feng, Youqing Luo

**Affiliations:** Key Laboratory for Silviculture and Conservation of Ministry of Education, Beijing Forestry University, Beijing, 100083, P.R. of China; Institute of Vegetables and Flowers, Chinese Academy of Agricultural Sciences, CHINA

## Abstract

*Ginkgo biloba* is a typical relic plant that rarely suffers from pest hazards. This study analyzed the pattern of *G*. *biloba* pest hazards in Beijing; tested the antifeedant activity of *G*. *biloba* extracts, including ginkgo flavonoids, ginkgolide, and bilobalide, against *Hyphantria cunea* larvae; determined the activities of glutathione transferase (GSTs), acetylcholinesterase (AChE), carboxylesterase (CarE) and mixed-functional oxidase (MFO), in larvae after feeding on these *G*. *biloba* secondary metabolites; and screened for effective botanical antifeedants in the field. In this study, no indicators of insect infestation were found for any of the examined leaves of *G*. *biloba*; all tested secondary metabolites showed significant antifeedant activity and affected the activity of the four larval detoxifying enzymes. Ginkgolide had the highest antifeedant activity and the most significant effect on the detoxifying enzymes (P<0.05). Spraying leaves with *G*. *biloba* extracts or ginkgolide both significantly repelled *H*. *cunea* larvae in the field (P<0.05), although the former is more economical and practical. This study investigated the antifeedant activity of *G*. *biloba* secondary metabolites against *H*. *cunea* larvae, and the results provide new insights into the mechanism of *G*. *biloba* pest resistance. This study also developed new applications of *G*. *biloba* secondary metabolites for effective pest control.

## Introduction

During the long-term co-evolution with insects, plants gained the ability to produce a plethora of secondary metabolites through accidental genetic mutation and genetic recombination. Secondary metabolites are irrelevant for normal plant growth, but have key roles for chemical defense against insects by killing, repelling, inhibiting the feeding, or hindering the growth of insects [1]. These secondary metabolites also have important effects on insects’ selection of host plants [2]. During co-evolution, plants developed a chemical defense system of secondary metabolites to prevent insect feeding, and insects adaptively developed detoxifying enzymes to defeat this plant chemical defense system [3].

*Ginkgo biloba* is a rare and endangered tree species unique to China, and the only living member of the *G*. *biloba* order of plants, which historically included at least seven genera as indicated by the fossil record. *G*. *biloba* has been widely cultured in China for at least 1,000 years, and all *G*. *biloba* cultured in other countries are derived from China, making it a typical cash crop [4]. *G*. *biloba* produces many important secondary metabolites, among which ginkgo flavonoids and ginkgolide are unique to this plant. These secondary metabolites accumulate to relatively high contents in *G*. *biloba*, and the levels may differ among different plant parts and in different seasons. Ginkgo flavonoids accumulate to the highest levels in tender leaves, and gingkolides have the highest levels during July to September [5–6].

*Hyphantria cunea* (Lepidoptera, Arctiidae) originated in North America. It is a major invasive species on the international quarantine list, and was first identified in China in 1979. These insects feed on the leaves of many plants, rapidly spread, and cause serious ecological disaster [7–8]. The detoxifying system of *H*. *cunea* includes glutathione transferase, carboxylesterase and mixed-functional oxidase, which have important roles in degrading plant secondary metabolites. Acetylcholinesterase is a key enzyme involved in synaptic conduction of insects, and is an important target for insecticides [9–10].

Botanical antifeedants are a class of compounds that inhibit insect feeding, although they do not directly kill insects. Most plant secondary metabolites that show antifeedant activity can be classed into the following four categories: sesquiterpene lactones, heterogeneous flavonoids, quassins, and limonoids. Some of these agents show only relative antifeedant activity within a certain period, but they do affect insect host selection. Botanical antifeedants are rapidly degraded after application, thereby causing little environmental impact [11].

*Ginkgo biloba* is a typical relic plant with strong resistance to various insects. Field observations of *G*. *biloba* rarely reveal any signs of pest damage. Sometimes, mild and sparsely distributed insect damage can be observed, but *G*. *biloba* rarely exhibits disastrous pest damage [12–14]. So, This study investigated the antifeedant activity of secondary metabolites that are unique to *G*. *biloba*. The results provide new insights into the mechanism underlying the strong resistance to insects, and show that application of *G*. *biloba* secondary metabolites provides effective antifeedant activity.

## Materials and Methods

### Antifeedant Agents and Source of Insects

*Ginkgo biloba* extracts contains approximately 25% ginkgo flavonoids and 5% ginkgolide. The ginkgo flavonoids used in this study contained quercetin, kaempferol, and isorhamnetin (Sigma-Aldrich, USA) at a ratio of 2:2:1 (w/v). The ginkgolide used in this study contained bilobalide and ginkgolide A/B/C (Sigma-Aldrich, USA) at a ratio of 5:2:2:1 (w/v).

Normal artificial diet and *H*. *cunea* egg mass were purchased from the Chinese Academy of Forestry. The *H*. *cunea* larvae grew, developed, and reproduced normally after feeding the normal artificial diet [15]. *H*. *cunea* larvae for indoor studies were all hatched from the same egg mass, and were starved for 8 hours before using in experiments.

### Analysis of Pest Feeding

This study was conducted in areas populated by *G*. *biloba* in Beijing City in June 2014. A total of 30 *G*. *biloba* trees were selected in each of 10 sample plots. Permission for each sample investigation was authorized by Beijing Municipal Bureau of Landscape and Forestry, and activities at each location were supervised by relevant staff from the department responsible. Three distinct classes rating tree health were established to analyze pest infestation (Table 1) [16]. Tree height, diameter, health status, and site and type of insect infestation were measured and recorded. The pest resistance percentage was calculated as the number of pest-free trees among the total number of trees in the plot.

**Table 1:** Classification of tree health based on the extent of pest feeding and the degree of damage to Ginkgo biloba.

Classification	Number of emerged pests or defecation holes	Proportion of leaves with symptoms of pest feeding
Class 1	0	0%
Class 2	1–5	1–50%
Class 3	>5	>50%

$$\text{Pest resistance percentage}=\frac{\text{Number of pest free trees}}{\text{Total number of trees in the plot}}\times 100$$

### Verification of Antifeedant Activity

To make the normal diet, feed powder (20 g), distilled water (80 ml), and agar power (1.6g) were mixed and heated to completely dissolve the agar. To make the test diet, *G*. *biloba* secondary metabolites were dissolved in 10 ml ethanol before mixing them with the normal diet at different concentrations. All the diets were poured into a rearing box (100ml, d = 5cm) and allowed to solidify. The resulting circular pie (δ = 2mm) was then cut into two equal semicircles for future use. Six other rearing boxes of the same shape was prepared, and a semicircular pie of the normal diet and six semicircular pies containing 4%, 8%, or 16% *G*. *biloba* extract, 2% flavonoids, 0.4% ginkgolides, or 0.2% bilogalide were placed in the boxes. Then 30 third-instar *H*. *cunea* larvae were put into each box, and the number of larvae choosing to feed on each of the semicircular pies in the boxes was recorded after 24h. These experiments were repeated five times [17–20].

### Detoxifying Enzyme Activity Assay

Circular diet pies containing 8% *G*. *biloba* extracts, 2% ginkgo flavonoids, 0.4% ginkgolide, 0.2% bilobalide, or no extra additive were prepared as described above, to feed 120 third-instar *H*. *cunea* larvae in each group. The activities of glutathione transferase, acetylcholinesterase, carboxylesterase and mixed function oxidase were measured at 12, 24, 36, and 48 h for each group of 30 larvae. Third-, fourth-, and fifth-instar *H*. *cunea* larvae were fed on a diet containing the above four *G*. *biloba* secondary metabolites, and the activities of the above detoxifying were measured at 24 h in each group of enzymes in each group of 30 larvae. These experiments were repeated five times.

Glutathione transferase (GSTs) activity was measured using Lee’s method. Briefly, the tissue sample was weighed, and 9 volumes of normal saline were added to produce a 10% tissue homogenate, which was then centrifuged at 12,000 rpm for 30 min at 4°C. The supernatant was considered as the enzyme solution. The enzyme reaction was measured in a cuvette containing 2.4 mL phosphate buffer (66 mmol/L, pH 7.0), 0.1 mL enzyme solution, 0.3 mL of 50 mmol/L glutathione (GSH), and 0.1 mL of 30 mmol/L 1-chloro-2,4-dinitrobenzene (CDNB), and changes in absorption at 340 nm were recorded. The measurement was repeated five times [21].

Acetylcholinesterase (AChE) activity was measured using a modified Ellman method. Briefly, the tissue sample was weighed, and 9 volumes of normal saline were added to produce a 10% tissue homogenate, which was then centrifuged at 3,500 rpm for 10 min at 4°C. The supernatant was collected for enzyme activity measurements. Supernatant (0.1 mL) and ATCh (0.1 mL) (a substrate of AChE) were mixed together and incubated at 30°C for 15min, and then 3.6 mL DTNB was added to terminate the reaction. Absorbance was measured at 412nm. The experiment was repeated five times [22–23].

Carboxylesterase (CarE) activity was measured using Asperen’s method. Briefly, the tissue sample was weighed, and 9 volumes of normal saline were added to produce a 10% tissue homogenate, which was then centrifuged at 12,000 rpm for 30 min at 4°C. The supernatant was collected and diluted 50× with phosphate buffer before using for enzyme activity measurement. The enzyme reactions contained 0.3 mL of the diluted supernatant, 0.7 mL of 0.04 mol/L phosphate buffer, and 5 mL substrate, which were mixed and incubated on a shaker for 30 min at 37°C. Then, 1 mL of chromogenic reagent was added, and the reactions were incubated for a further 30 min at room temperature, followed by measurement of absorbance at 600 nm. The experiment was repeated five times. Known concentrations of α-naphthol were used to obtain a standard curve [24].

Mixed function oxidase (MFO) activity was measured using Yu and Nguyen’s method. Tissue samples were weighed and homogenized in 200μl ice-cold homogenization buffer (10% glycerol in 0.1 mol/L phosphate buffer, pH7.5). Then the homogenate was centrifuged at 4°C, 12000r/min for 10min, and the supernatant was centrifuged again. The final supernatant was collected for enzyme activity measurements. Paranitroanisole was added to the supernatant as a substrate for MFO, and the para-nitrophenolate produced was used to calculate MFO activity. The activity of each sample was measured five times [25].

Protein content was measured according to the Bradford’s method using bovine serum albumin as the standard.

### Leaf-Disc Assay

*Ginkgo biloba* extracts, ginkgo flavonoids, ginkgolide, and bilobalide were dissolved in 20 mL absolute alcohol:water [1:7 (v/v)] solution in a concentration series (Table 2). Fresh *Fraxinus americana* leaf discs (1.5 cm diameter) were prepared with a drilling machine from healthy and pesticide-free leaves, and were soaked in the secondary metabolite solutions or alcohol: water [1:7 (v/v)] (as a control) for 10 sec. Four of the treated leaf discs were placed into each empty rearing box (100 ml, d = 5cm) to feed 30 third-instar *H*. *cunea* larvae for 24 h. Then, the leaves were removed from the boxes and placed on 1×1 mm graph paper to measure the eaten area by counting the grids. The experiment was repeated five times, and the antifeedant rate was calculated [26].

**Table 2:** Concentration series for four *Gingko biloba* secondary metabolites.

Secondary metabolite	High(g/L)	Medium(g/L)	Low(g/L)	Control group(g/L)
GBE	50	25	12.5	0
GF	12.5	6.25	3.125	0
GL	2.5	1.25	0.625	0
BB	1.25	0.625	0.3125	0

GBE, *Ginkgo biloba* extract; GF, gingko flavonoids; GL, ginkgolide; BB, bilobalide.

$$\text{Antifeedant rate }=\frac{\text{Eaten area of the control group }-\text{ Eaten area of the test group}}{\text{Eaten area of the control group}}\times 100$$

### Field Antifeedant Test

For field antifeedant tests, 2 g of *G*. *biloba* extracts, 0.5g of ginkgo flavonoids, 0.1g ginkgolide and 0.05g of bilobalide were respectively dissolved in 10 mL absolute alcohol, mixed with seven volumes of water, and stored in a 100 mL plastic watering can that was properly labeled. Alcohol: water [1:7 (v/v)] was used as the vehicle control. The tests included 183 branches of 24 *Salix* trees with a diameter of 12–21 cm in the suburban area of Tianjin, China, an area of 0.08 km^2^ where Salix trees suffered severe damage from *H*. *cunea* larvae infestation in August 2015 (Table 3). Photographs were taken to record the insect feeding situation. The five solutions (four secondary metabolites and one control) were sprayed on infested leaf surfaces 3–5 times, and the larval feeding situation was recorded by photographing again from the same perspective 5 h later. The repelling rate was calculated by comparing the number of larvae on the photographs taken before and after spraying [27].

**Table 3:** Instars of *Hyphantria cunea* larvae infesting branches treated with *Ginkgo biloba* secondary metabolites.

Instar	1st	2nd	3rd	4th	5th	Total
GBE	10	11	6	6	6	39
GF	13	9	7	5	5	39
GL	15	10	8	5	6	44
BB	9	9	7	6	5	36
CG	5	5	5	5	5	25

GBE, *Ginkgo biloba* extract; GF, gingko flavonoids; GL, ginkgolide; BB, bilobalide; CG, control group.

$$\text{Repelling rate }=\frac{\text{Number of larvae before }-\text{ Number of larvae after}}{\text{Number of larvae before}}\times 100$$

### Data Analysis

Variance analysis and data processing were performed using SPSS 17.0 or Prism 6.0. One-way ANOVA followed by least significant difference test (LSD, at a level of statistical significance of P = 0.05) were used to analyze the percentage of larva feeding on a given diet or food source, the detoxifying enzyme activities, antifeedant rate, and repelling rate. All of the data expressed as percentages were arcsine transformed and analyzed using SPSS 17.0.

## Results

### Pest Damage to *G*. *biloba* Trees in Beijing City

The average height of *G*. *biloba* trees in the 10 sample plots was 6.1–15.1 m, and the average diameter was 13.3–49.9 cm. Symptoms of pest feeding on the trees are summarized in Table 4. All pest feeding symptoms were confined to the trunk, and no symptoms were observed on leaves. Approximately 93.3% or more trees in the selected plots were free of insect damage in the same year. Only six *G*. *biloba* trees in Plots A, C, F, and G were damaged by *Holcocerus insularis* Staudinger (Lepidoptera, Cossidae) larvae, and only two trees were rated as Class 3 damage. No damage by any insect was found in the other six plots.

**Table 4:** Evaluation of pest feeding on *Ginkgo biloba* in Beijing City.

Location	Samplesize	Averageheight(m)	Average diameter(cm)	Class 1	Class 2	Class 3	Proportions without pest(%)	Affected part of tree	Pest species
A	30	15.1	49.9	28	1	1	93.3	Trunk	H. insularis
B	30	12.1	32.7	30	0	0	100.0		
C	30	11.7	31.3	29	1	0	96.7	Trunk	H. insularis
D	30	11.4	30.3	30	0	0	100.0		
E	30	10.8	28.7	30	0	0	100.0		
F	30	10.5	28.5	28	1	1	93.3	Trunk	H. insularis
G	30	10.1	27.9	29	1	0	96.7	Trunk	H. insularis
H	30	8.8	24.7	30	0	0	100.0		
I	30	8.7	21.0	30	0	0	100.0		
J	30	6.1	13.3	30	0	0	100.0		

A, Peking University; B, Diaoyutai State Guesthouse; C, Ditan Park; D, Tsinghua University; E, Changping Zhangge village; F, Beijing Forestry University; G, Beijing Botanical Garden; H, Olympic Forest Park; I, Chaoyang District, Sanlitun West Fifth Street; J, Haidian Minzhuang Road.

### Antifeedant Activity of Different *G*. *biloba* Secondary Metabolites against *H*. *cunea* Larvae

**Fig 1:**
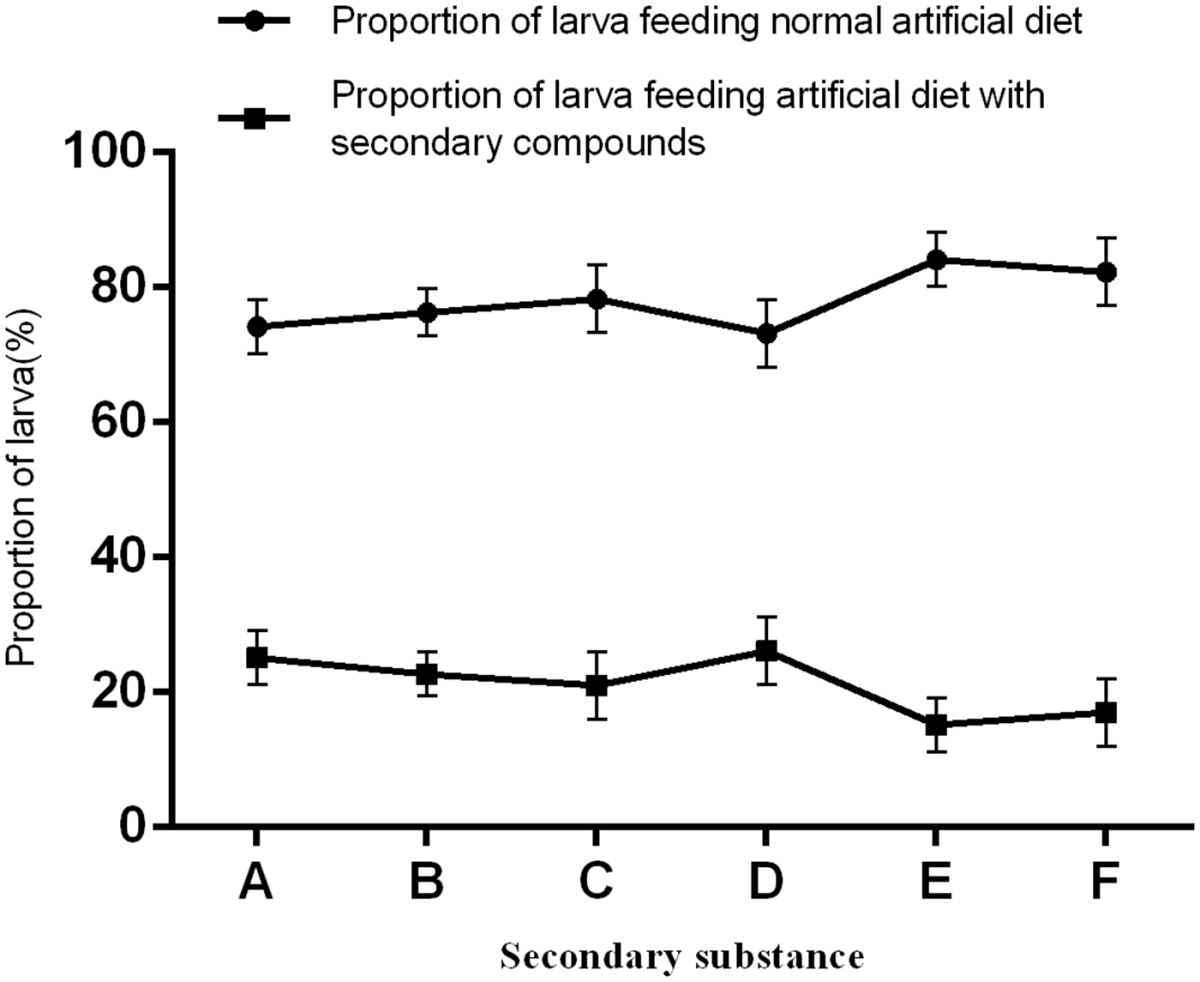
Analysis of artificial diet choice by *Hyphantria cunea* larvae using diets containing different types of *Gingko biloba* secondary materials. A: 4% extract of ginkgo biloba; B: 8% extract of ginkgo biloba; C: 16% extract of ginkgo biloba; D: 2% gingko flavonoids; E: 0.4% ginkgolide; F: 0.2% bilobalide.

The two curves (Fig 1) show that the proportion of *H*. *cunea* larva feeding on the diet containing *G*. *biloba* secondary metabolites was significantly lower than the number feeding on the normal diet (F = 36.579; df = 5, 48; P<0.0001). *Ginkgo biloba* extracts significantly repelled *H*. *cunea* larvae (F = 6.492; df = 2, 12; P = 0.023), and the effect was stronger at higher extracts concentrations (Fig 1A–1C). The concentration of ginkgo flavonoids, ginkgolide, and bilobalide were defined according to 8% *G*. *biloba* extracts. Comparing these results with those of Fig 1D–1F and 1B, it can be concluded that ginkgo flavonoids, ginkgolide, and bilobalide significantly repel *H*. *cunea* larvae (F = 13.585; df = 3, 16; P = 0.009), and ginkgolide and bilobalide show stronger repelling effects than that of the 8% *G*. *biloba* extracts.

### Effects of *G*. *biloba* Secondary Metabolites on Detoxifying Enzyme Activities

**Fig 2:**
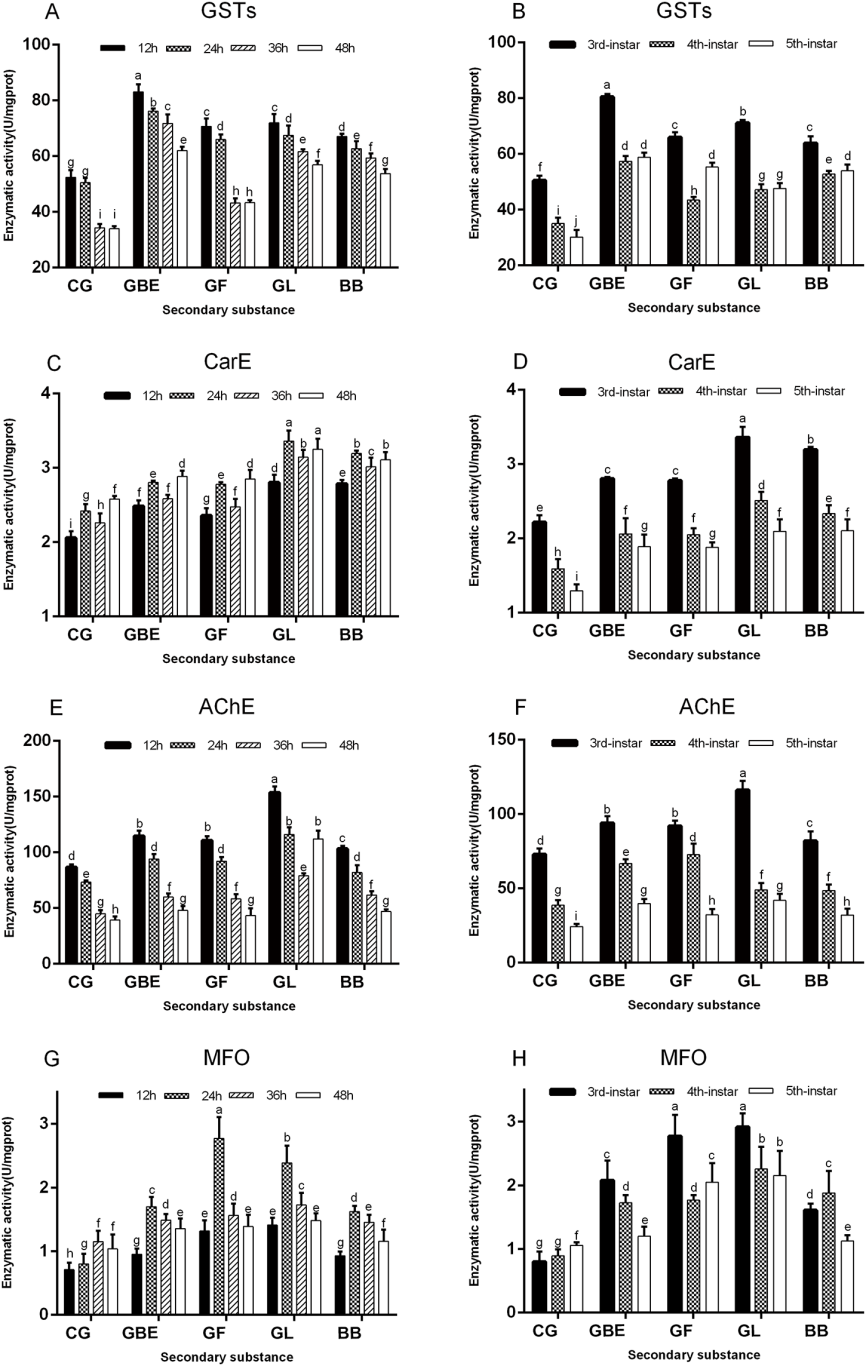
Analysis of the enzyme activities of four detoxifying enzymes of *Hyphantria cunea* larvae fed artificial diets with *Gingko biloba* secondary metabolites. (A)The enzymatic activity of GSTs were measured in different feeding time. (B)The enzymatic activity of GSTs were measured in different larvae instar. (C)The enzymatic activity of CarE were measured in different feeding time. (D)The enzymatic activity of CarE were measured in different larvae instar. (E)The enzymatic activity of AChE were measured in different feeding time. (F)The enzymatic activity of AChE were measured in different larvae instar. (G)The enzymatic activity of MFO were measured in different feeding time. (H)The enzymatic activity of MFO were measured in different larvae instar. EGB: extract of ginkgo biloba; GF: gingko flavonoids; GL: ginkgolide; BB: bilobalide; CG: contral group. Different letters above bars indicate significant differences (P<0.05).

The activities of four detoxifying enzymes of *H*. *cunea* larvae increased after feeding on diet containing *G*. *biloba* secondary metabolites, but the extent to which the activities varied differed because of differences in the detoxification mechanisms of the enzymes. GSTs activity was affected most strongly by *G*. *biloba* extracts in the Fig 2A (F = 37.965; df = 4, 80; P<0.0001) and B (F = 40.861; df = 4, 60; P<0.0001), CarE activity responded more rapidly to ginkgolide and bilobalide in the Fig 2C (F = 67.249; df = 4, 60; P<0.0001) and D (F = 30.852; df = 4, 80; P = 0.001), and AChE activity was affected most strongly by ginkgolide in the Fig 2E (F = 30.327; df = 4, 80; P = 0.001) and F (F = 33.985; df = 4, 60; P<0.0001), MFO activity responded more rapidly to gingko flavonoids and ginkgolide in Fig 2G (F = 33.416; df = 4, 80; P<0.0001) and H (F = 42.589; df = 4, 60; P<0.0001). GSTs (F = 37.965; df = 4, 80; P<0.0001) and AChE (F = 30.327; df = 4, 80; P<0.0001) activities were relatively higher in the first 24 h (Fig 2A and 2E), CarE activity was relatively higher at 24 and 48 h (Fig 2C) (F = 30.852; df = 4, 80; P = 0.001) and MFO activity was relatively higher at 24 h (Fig 2G) (F = 33.416; df = 4, 80; P<0.0001). GSTs (F = 40.861; df = 4, 60; P<0.0001), CarE (F = 67.249; df = 4, 60; P<0.0001), AChE (F = 33.985; df = 4, 60; P<0.0001)and MFO (F = 42.589; df = 4, 60; P<0.0001) activities were the highest for third-instar larvae at the same time point (Fig 2B, 2D, 2F and 2H).

### Antifeedant Activity of *G*. *biloba* Secondary Metabolites against *H*. *cunea* Larvae as Assessed by the Leaf-Disc Assay

**Fig 3:**
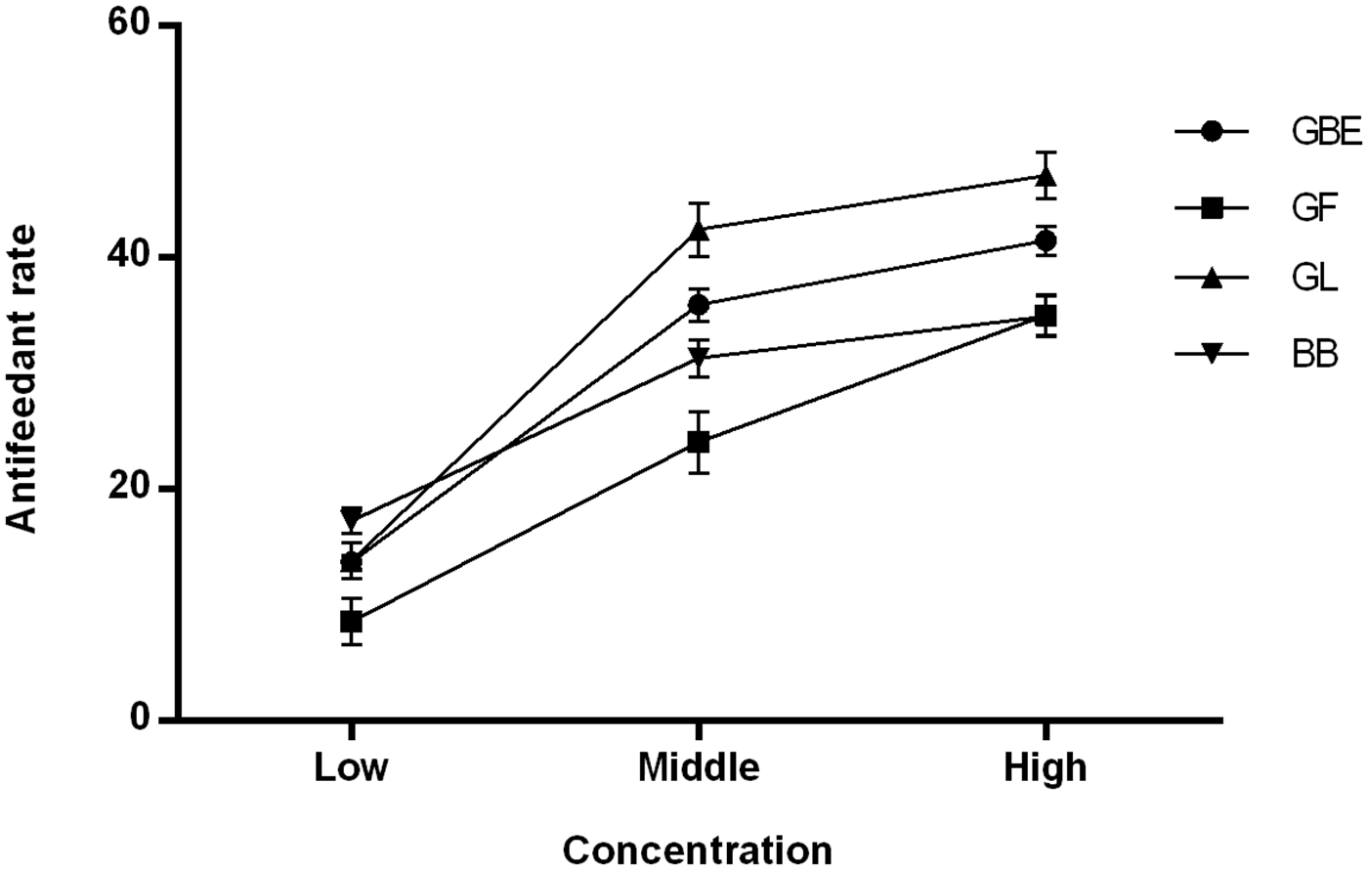
Antifeedant rate of leaf discs soaked by treated with *Gingko biloba* secondary materials. EGB: extract of ginkgo biloba; GF: gingko flavonoids; GL: ginkgolide; BB: bilobalide.

The leaf-disc assay results show that all tested *G*. *biloba* secondary metabolites have antifeedant activity against *H*. *cunea* larvae (Fig 3). The antifeedant activity of all four secondary metabolites showed concentration-dependent larval responses (F = 22.986; df = 3, 48; P = 0.002). At low concentrations, the antifeedant activities of all four secondary metabolites were not prominent. At medium and high concentrations, all four secondary metabolites showed strong antifeedant activity. The antifeedant efficiency in descending order was ginkgolide > *G*. *biloba* extracts > bilobalide > ginkgo flavonoids (F = 18.773; df = 3, 32; P = 0.003).

### Antifeedant Activity of *G*. *biloba* Secondary Metabolites against *H*. *cunea* Larvae in the Field

**Fig 4:**
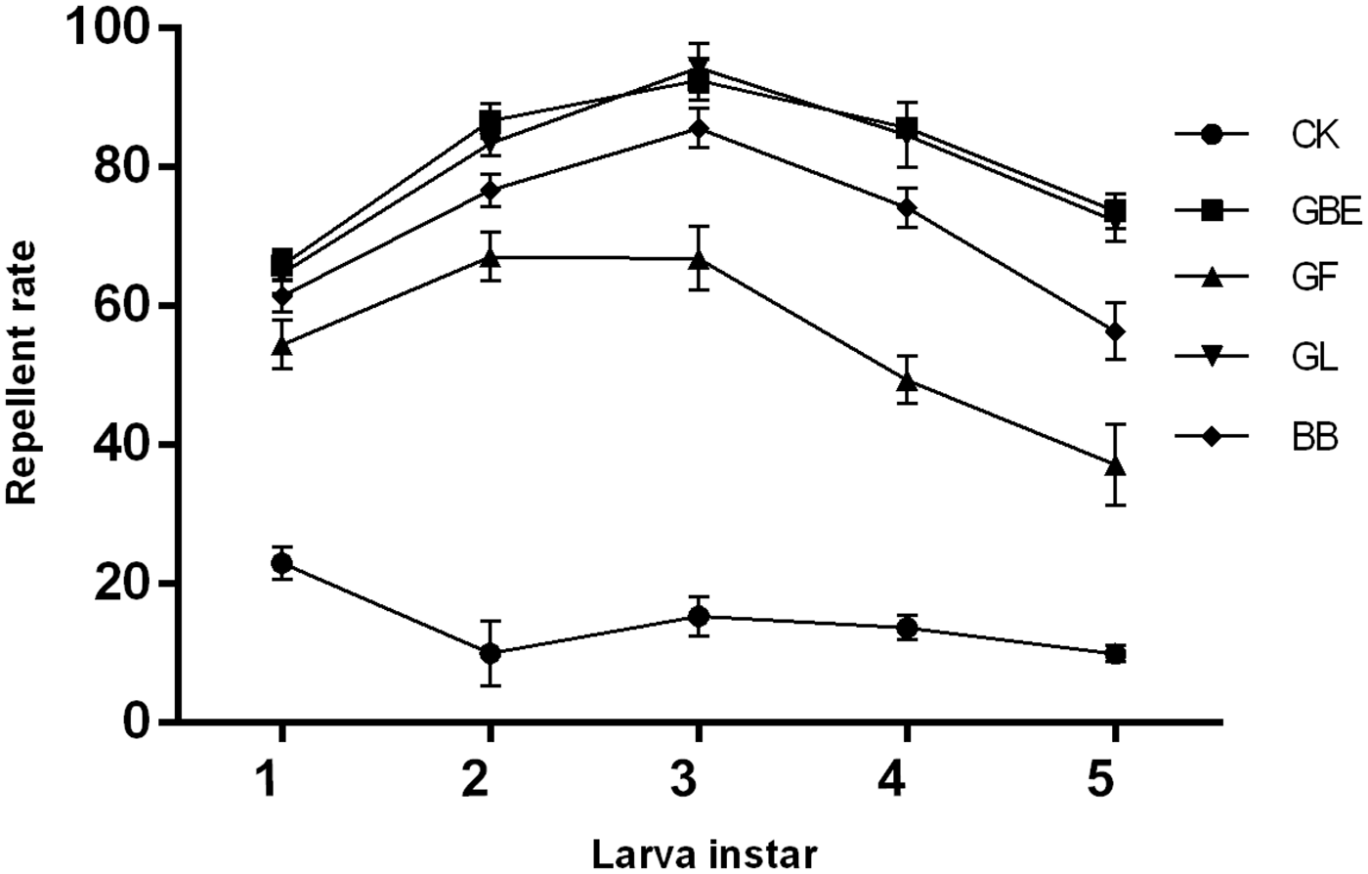
Repellent rate of medicament with *Gingko biloba* secondary metabolites to *Hyphantria cunea* larvae. EGB: extract of ginkgo biloba; GF: gingko flavonoids; GL: ginkgolide; BB: bilobalide; CG: control group.

All four tested secondary metabolites significantly repelled *H*. *cunea* larvae compared with that of the vehicle control(Fig 4) (F = 58.175; df = 4, 100; P<0.0001). In all larval instars, ginkgolide and *G*. *biloba* extracts showed the strongest repelling effects, whereas ginkgo flavonoids had the weakest. Third-instar larvae were the most sensitive to *G*. *biloba* secondary metabolites, whereas newly hatched and aged larvae showed relatively lower sensitivities to these agents (F = 28.276; df = 4, 100; P = 0.001).

## Discussion and Conclusions

*Ginkgo biloba* is a typical relic plant that rarely exhibits severe pest infestations, and reports about pest hazards to *G*. *biloba* leaves are rare. We tested the antifeedant activity of *G*. *biloba* extracts and isolated secondary metabolites, including ginkgo flavonoids, ginkgolide, and bilobalide, against *H*. *cunea* larvae. The results showed that all four preparations conferred significant antifeedant activity in leaf-disc assays and in artificial diets, and the activities of larval detoxifying enzymes significantly increased after feeding on diet containing these substances. These preparations also significantly repelled *H*. *cunea* larvae in field tests. Ginkgolide, which is unique to *G*. *biloba*, showed the strongest antifeedant activity, the strongest effect on detoxifying enzyme activity, and the strongest repelling effect in field tests. Studies have shown that ginkgolides can be detected in various tissues of *G*. *biloba*. Their contents ranged from high to low in the order leaf > root > stem [28]. No sign of insect feeding was observed on leaves of *G*. *biloba*, but fresh and past-year emergence holes of *H*. *insularis* Staudinger were observed on the stem, which may be due to differences in ginkgolide concentrations in these tissues. The ginkgolide used in this study was a mixture of four monomers, and the efficacy of the major lactone, bilobalide, alone also showed significant antifeedant efficacy toward *H*. *cunea* larvae. All of these monomers are secondary metabolites unique to *G*. *biloba*, which highlights the antifeedant activity of secondary metabolites produced by relic plants that co-evolved with insects [29].

MFO is one kind of oxidase system, and can carry on the oxidation of foreign substances in the insect body, GSTs can inactivate exogenous and endogenous toxin molecules and convert them into water-soluble compounds, CarE can convert compounds containing ester compounds into alcohol and acid [30–34]. *Ginkgo biloba* secondary metabolites affected the activities of glutathione transferase, carboxylesterase, acetylcholinesterase and mixed-functional oxidase activity, which is involved in the nervous system. These results suggest that the antifeedant activity of *G*. *biloba* secondary metabolites against *H*. *cunea* larvae may be multifactorial.

Under field conditions, *G*. *biloba* secondary metabolites do not kill the insects; rather, the strong antifeedant activity affects plant host selection by the insects. Larvae rapidly move away after spraying preparations containing *G*. *biloba* secondary metabolites on *Salix* branches damaged by *H*. *cunea* larvae. This result indicates the strong repelling effect conferred by these agents [35]. Although ginkgolide showed similar repelling effect as that of *G*. *biloba* extracts, it is more practical and economical to use preparations containing *G*. *biloba* extracts.

Plants can be classified into different families, genera, and species. After long-term evolution and specialization, insects feeding on certain categories of plants developed detoxification mechanisms to infest more effectively plants that produce these metabolites. Secondary substances produced by non-host plants show antifeedant effects on insects. *G*. *biloba* is a relic plant, with no parallel of the same family or genus, and plays host to a few insects. Its unique secondary metabolites show significant antifeedant and repelling effects, but they are not toxic to *H*. *cunea* larvae, so insects will not normally develop resistance to these metabolites after genetic selection. The unique secondary metabolites of G. biloba undergo rapid degradation after spraying in the wild, and thus have little impact on the environment. Beside G. biloba, some other plant species are unique to the family and genus; thus, the unique secondary metabolites of G. biloba could actually represent a large group of potential plant protection agents. Secondary metabolites of different relic plants can be mixed to widen the anti-insect spectrum, or alternatively, the genes encoding these secondary metabolites could be introduced into other plant to generate new resistant variants [36].

The long-term co-evolution of insects and plants promoted the development of plant secondary metabolites, which emerged to have key roles in chemical defense and insect resistance. *G*. *biloba* survived from the quaternary glaciation period at least partly owing to its strong pest resistance conferred by its unique secondary metabolites. *G*. *biloba* has a rich array of secondary metabolites that may accumulate and decline with changing seasons, so that the peak content of chemical agents largely overlaps with seasonal peaks in insect activity [28]. This study presented evidence for the antifeedant and repelling effects of *G*. *biloba* secondary metabolites. The results provide new insights into the function of unique *G*. *biloba* secondary metabolites, and a new understanding of why *G*. *biloba* is largely free of insect pests [37].

## References

[1] Zhu L, Gu D. The adaptive strategies of insects to Plant Alleochemicals. Chinese Journal of Ecology. 2000; 19(3): 36–45.

[2] Wang CZ, Qin JD. Insect-plant co-evolution: multitrophic interactions concerning *Helicoverpa* species. Chinese Bulletin of Entomology. 2007; 44(3): 311–319.

[3] Peng L, Yan Y, Liu WX, Wan FH, Wang JJ. Counter-defense mechanisms of phytophagous insects towards plant defense. Acta Entomologica Sinica. 2010; 53(5): 572–580.

[4] Li L, Zhang GF, Wang R, Sun G, Zhao MS. Life table of natural *Ginkgo biloba* population in tianmu mountain nature reserve. Chinese Journal of Ecology. 2011; 30(1): 53–58.

[5] Van Beek TA, Montoro P. Chemical analysis and quality control of *Ginkgo biloba* leaves, extracts, and phytopharmaceuticals. Journal of Chromatography A. 2009; 1216(11): 2002–2032. 10.1016/j.chroma.2009.01.013 19195661

[6] Li J, Chase HA. Use of expanded bed adsorption to purify flavonoids from *Ginkgo biloba* l. Journal of Chromatography A. 2009; 1216(50): 8759–8770. 10.1016/j.chroma.2009.03.002 19321174

[7] Rong JI, Xie BY, Li XH, Gao ZX, Li DM. Research progress on the invasive species, *Hyphantria cunea*. Entomological Knowledge. 2003; 40(1): 13–18.

[8] Zhang YL, Wu SA, Guo WX, Chen HZ. Research progress on biological control of fall webworm (Hyphantria cunea Drury) in china. Hebei Journal of Forestry & Orchard Research. 2008; 23(1): 70–77.

[9] Van Leeuwen T, Van Pottelberge S, Tirry L. Comparative acaricide susceptibility and detoxifying enzyme activities in field-collected resistant and susceptible strains of *Tetranychus urticae*. Pest Management Science. 2005; 61(5): 499–507. 15657956 10.1002/ps.1001

[10] Wang R, Sun Y, Liang X, Song Y, Yijuan SU, Keyuan ZS et al Effects of six plant secondary metabolites on activities of detoxification enzymes in *Spodoptera litura*. Acta Ecologica Sinica. 2012; 32(16): 5191–5198.

[11] Li SQ, Fang YL, Zhang ZN. Studies and applications of botanical insect antifeedants. Entomological Knowledge. 2005; 42(5): 491–496.

[12] Liang HL, Wei X, Feng LI, Jiang YS. Investigation and control measures of ginkgo diseases and insect pests. Acta Agriculturae Jiangxi. 2008; 20(5): 49–51.

[13] Mohanta TK, Occhipinti A, Zebelo SA, Foti M, Fliegmann J, Simone B et al *Ginkgo biloba* responds to herbivory by activating early signaling and direct defenses. Plos One. 2012; 7(3): 504–504. 10.1371/journal.pone.0032822 PMC3308967 22448229

[14] Pszczolkowski MA, Kevin D, Samantha S, Brian C, Brown JJ. Effects of *ginkgo biloba* constituents on fruit-infesting behavior of *Codling moth* (cydia pomonella) in apples. J.agric.food Chem. 2011; 59(20): 10879–10886. 10.1021/jf202386c 21905729

[15] Zhang YA, Wang YZ, Xu BM, Qu LJ. The method of artificial feeding and passage of *Hyphantria cunea* and its larvae feed. CN, CN 101228853 A. 2008.

[16] Batala E, Tsitsoni T. Street tree health assessment system: a tool for study of urban greenery. International Journal of Sustainable Development and Planning. 2009; 4(4): 345–356.

[17] Xu D, Huang Z, Cen YJ, Chen Y, Freed S, Hu XG. Antifeedant activities of secondary metabolites from *Ajuga nipponensis* against adult of striped flea beetles, *Phyllotreta striolata*. Journal of Pest Science. 2009; 82(2): 195–202.

[18] Á. A. Jiménez R., L. D. Rodríguez R., W. Murillo A., J. J. Méndez A., & E. A Rueda L.. (2013). Antifeedant activity of secondary metabolites of citrus waste on Spodoptera frugiperda (lepidoptera: noctuidae). Revista Colombiana De Entomologiía, 39(1), 113–119.

[19] Masanori M, Kumiko T, Sachiko N, Takayoshi O, Ayako N, Koichiro K. Insect antifeedant activity of flavones and chromones against *Spodoptera litura*. Journal of Agricultural & Food Chemistry. 2003; 51(2): 389–393. 12517100 10.1021/jf025627a

[20] Akhtar Y, Isman MB. Comparative growth inhibitory and antifeedant effects of plant extracts and pure allelochemicals on four phytophagous insect species. Journal of Applied Entomology. 2004; 128(1): 32–38.

[21] Pan YF, Meng JY, Zhang XY, Zhou XM, Lei CL. Effects of host plants on insecticide susceptibility of bean pod borer, *Maruca testulalis*, and activity of its detoxification enzyme. Chinese Bulletin of Entomology. 2006; 43(4): 496–500.

[22] Dai Y, Wang X, Zhang Y, Wu Q, Xu B, Xie W et al Induction effects on the detoxification enzymes of Tetranychus urticae from different host plants. Plant Protection. 2012; 38(2): 79–82.

[23] Yin F, Chen HY, Li ZY, Lin QS, Hu ZD, Zhang DY et al Changes in susceptibility to chlorantraniliprole and detoxification enzymes of *Plutella xylostella* feeding on different host plants. Chinese Journal of Applied Entomology. 2013; 50(5): 1335–1340.

[24] Dong JF, Zhang JH, Wang CZ. Effects of plant allelochemicals on nutritional utilization and detoxication enzyme activities in two *Helicoverpa* species. Acta Entomologica Sinica. 2002; 45(3): 296–300.

[25] Liao YZ, Yan SC, Cao CW, Liu D. Effect of methoxyfenozide on activities of detoxifying enzymes and expression of proteins in *Lymantria dispar* larvae. Scientia Silvae Sinicae, 2012, 48(8):99–105.

[26] Hu X, Yan SC, Lu YF, Liu D. Antifeedant activity of the secondary metabolic compounds of yew against *lymantria dispar* L larvae. Journal of Beijing Forestry University. 2011; 33(5): 151–154.

[27] Prisila M, Regina M, Stevenson PC, Patrick N, Kelvin M, Steven RB. Extracts from field margin weeds provide economically viable and environmentally benign pest control compared to synthetic pesticides. Plos One. 2015; 10(11). 10.1371/journal.pone.0143530 PMC4658159 26599609

[28] Leng PS, Wang TH, Su SC, Jiang XN, Wang SS. Seasonal changes of flavonol glycosides and terpene lactone contents of *ginkgo biloba* L. Journal of Plant Resources & Environment. 2001; 10(3): 15–18.

[29] Sang-Sup SO, Hwang TH, Akram W, Choi JK, Lee JJ. Insecticidal activities of ginkgo biloba seed coat extracts against Spodoptera exigua (lepidoptera: noctuidae). Entomological Research. 2012; 42(42): 158–162.

[30] Namountougou M, Simard F, Baldet T, Diabate A, Ouedraogo JB, Martin T et al Multiple Insecticide Resistance in *Anopheles gambiae*. l. Populations from Burkina Faso, West Africa. Plos One. 2012; 7(11). 10.1371/journal.pone.0048412 PMC3506617 23189131

[31] Silva AX, Bacigalupe LD, Luna-Rudloff M, Figueroa CC. Insecticide resistance mechanisms in the green peach sphid *Myzus persicae* (Hemiptera: Aphididae) I: A Transcriptomic SurveY. Plos One. 2012; 7(6). 10.1371/journal.pone.0036810 PMC3369902 22685539

[32] Fuentes-Contreras E, Reyes M, Barros WB. Evaluation of azinphos-methyl resistance and activity of detoxifying enzymes in *codling moth* (lepidoptera: tortricidae) from central chile. Journal of Economic Entomology. 2007; 100(2): 551–556(6). 17461082 10.1603/0022-0493(2007)100[551:eoaraa]2.0.co;2

[33] Kamila D, Blythe MJ, Sunir M, Genereux DP, Alessandro G, Paolo P et al Transient exposure to low levels of insecticide affects metabolic networks of honeybee larvae. Plos One. 2013; 8(8). 10.1371/journal.pone.0068191 PMC3699529 23844170

[34] Tang R, Zhang JP, Zhang ZN. Electrophysiological and behavioral responses of male fall webworm moths (*Hyphantria cunea*) to herbivory-induced mulberry (*Morus alba*) leaf volatiles. Plos One. 2012; 7(11). 10.1371/journal.pone.0049256 PMC3498160 23166622

[35] Yang ZQ, Zhang YA. Researches on techniques for biocontrol of the fall webworm, *Hyphantria cunea*, a severe invasive insect pest to china. Chinese Bulletin of Entomology. 2007; 44(4): 465–471.

[36] Wei H, Hou YM, Yang G, You MS. Repellent and antifeedant effect of secondary metabolites of non-host plants on *Plutella xylostella*. The journal of applied ecology, 2004, 15(3): 473–476. 15228000

[37] Lsman M. Botanical insecticides, deterrents, and repellents in modern agriculture and an increasingly regulated world. Annual Review of Entomology. 2005; 51: 45–66. 10.1146/annurev.ento.51.110104.151146 16332203

